# Influence of Transcranial Direct Current Stimulation on Psychomotor Symptoms in Major Depression

**DOI:** 10.3390/brainsci10110792

**Published:** 2020-10-29

**Authors:** Djamila Bennabi, Nicolas Carvalho, Ambra Bisio, Juliana Teti Mayer, Thierry Pozzo, Emmanuel Haffen

**Affiliations:** 1 Service de Psychiatrie de l’Adulte, Centre Hospitalier Universitaire de Besançon, 25030 Besançon CEDEX, France; nic.carvalh@gmail.com (N.C.); jtetimayer@chu-besancon.fr (J.T.M.); emmanuel.haffen@univ-fcomte.fr (E.H.); 2 Centre Expert Dépression Résistante FondaMental, Centre Hospitalier Universitaire de Besançon, 25030 Besançon CEDEX, France; 3 Centre d’Investigation Clinique, INSERM CIC 1431, Centre Hospitalier Universitaire de Besançon, 25030 Besançon CEDEX, France; 4 Laboratoire de Neurosciences Intégratives et Cliniques EA 481, Université de Bourgogne Franche-Comté, 19 rue Ambroise Paré, 25000 Besançon, France; 5 Department of Experimental Medicine, Section of Human Physiology and Centro Polifunzionale di Scienze Motorie, University of Genoa, Viale Benedetto XV 3, 16132 Genoa, Italy; ambra.bisio@unige.it; 6 INSERM U1093-Cognition, Action et Plasticité Sensorimotrice, Université de Bourgogne Franche-Comté, 21078 Dijon, France; thierry.pozzo@u-bourgogne.fr; 7 IIT@UniFe Center for Translational Neurophysiology of Speech and Communication, Istituto Italiano di Tecnologia, Via Fossato di Mortara, 17–19, 44121 Ferrara, Italy

**Keywords:** transcranial direct current stimulation, psychomotor symptoms, retardation, major depressive disorder

## Abstract

Background: Transcranial direct current stimulation (tDCS) applied to the left dorsolateral prefrontal cortex (dlPFC) might be a promising treatment strategy for depression. As disturbances in psychomotor activity are one of the key features of unipolar depression are, we aimed to evaluate the behavioral effects of ten tDCS sessions over a 5-day period on psychomotor retardation in depressed patients. Methods: Twenty-three treatment-resistant depressed patients received either active or sham anodal tDCS to the left dorsolateral prefrontal cortex (2 mA, 10 sessions over 1 week). Psychomotor functioning was registered by means of observer ratings (Salpêtrière Retardation Rating Scale—SRRS) and objective measures (kinematical analysis of movements, automatic imitation). Results: tDCS sessions resulted in improvements on SRRS scores, although active tDCS was not significantly superior to sham tDCS on the kinematical parameters. Furthermore, no general additional antidepressant effect of tDCS was observed. The relatively small sample size and the short periods of observation should be considered when interpreting these results. Conclusion: tDCS did not induce a clinically relevant effect on psychomotor function in active and sham stimulation groups.

## 1. Introduction

The worldwide spread of depressive disorders and associated comorbidities as well as their complex etiology and inter-individual variability in response to pharmacological interventions demand the development of new therapeutic strategies [1]. Non-invasive brain stimulation techniques have emerged as therapeutic options, guided by the emerging knowledge of mood-regulating systems. Among them, transcranial direct current stimulation (tDCS) has undergone intensive research over the past decade due to its favorable safety–feasibility profile and its ability to generate functional changes in resting membrane potential and cerebral blood flow [2]. A weak direct current of 1–2 mA is applied to generate regional changes in cortical excitability, which, depending on the duration and the polarity, can last for several minutes up to a few hours after stimulation [3]. Whereas the neuronal effects during tDCS are characterized by a shift of membrane potentials in cortical neurons that lead to a modification in the regional neuronal activity, sustainable effects seem to be mediated by bidirectional modifications of post-synaptic connections similar to long-term potentiation and long-term depression, occurring through N-methyl-D-aspartate (NMDA)-dependent mechanisms [4].

Clinical trials involving depression have typically applied anodal tDCS to the left dorsolateral prefrontal cortex (dlPFC) with the cathode placed over the contralateral cortex or the right supraorbital area to regulate cortical excitability of this area and restore the premorbid functional balance between the two hemispheres [5]. A meta-analysis of randomized controlled trials (RCTs) has found that active tDCS was superior to sham stimulation, with its effect size (B coefficient = 0.35) being comparable to that of repetitive transcranial magnetic stimulation (rTMS) and antidepressant medication in primary care [6]. Nonetheless, the percentage of responders remains suboptimal, and the clinical profile of the patients in whom tDCS may achieve its antidepressant effects needs to be clarified. Most tDCS studies focus on the reduction in mood symptoms in major depressive disorder (MDD), but the effects of tDCS on psychomotor functioning have been rarely explored [5].

Notwithstanding, psychomotor retardation (PMR) is recognized as a core feature of MDD and considered a good criterion to predict the therapeutic effect of pharmacological and non-pharmacological interventions, such as rTMS and electroconvulsive therapy [7]. The neurobiological process underlying PMR in MDD includes functional deficits in the left dlPFC, suggesting an important role of this cortical area on psychomotor functioning [8,9,10]. Therefore, given the possible neurobiological and clinical implications, a thorough investigation of the psychomotor effects of tDCS in MDD is warranted.

In healthy subjects, many studies have demonstrated a positive enhancement of neuromuscular performance. In MDD, a limited number of studies have already examined the impact of tDCS on psychomotor performance. In two consecutives RCTs, Loo et al. [11,12] failed to predict the response to anodal tDCS using the CORE index—a scale specifically designed to quantify the degree of psychomotor impairment—while they obtained positive results on depressive symptomatology after active versus sham stimulations. In a sample of 64 depressed patients treated with 15 sessions of anodal tDCS, Alonzo et al. [13] performed a factor analysis of the Montgomery and Asberg Depression Rating Scale (MADRS) and found a significant improvement in retardation symptoms. Recently, D’Urso and colleagues [14] published an interesting paper examining clinical indicators of optimal response to tDCS. In a six factors model of the Hamilton Depression Rating Scale (HDRS), the “retardation” factor displayed a considerable decrease after tDCS treatment and resulted as a predictor of an acute antidepressant effect.

Given the limited number of studies on this matter and their inconsistent results, this trial aimed to further investigate the psychomotor effects of tDCS in MDD by applying a simple imitation paradigm. This task is an objective and reliable method to assess motor activity, and it represents a promising and innovative manner to investigate the kinematic features of movements in natural conditions and in perception–action coupling [15,16,17]. It is considered a rater-independent and more precise and objective measurement method than the subjective rating scales. Moreover, this task has been previously used in further research of psychomotor symptoms, highlighting a global slowness of movements and a motor inhibition deficiency in MDD patients in comparison with healthy volunteers [18].

Hence, this study explores the psychomotor effects of anodal tDCS applied over the left dlPFC in a sample of treatment-resistant MDD patients through observer ratings and objective measures of psychomotor performance. The effect was assessed after 10 sessions spread over a period of 1 week of treatment. Data were collected as part of a recent randomized, double-blind, sham-controlled trial investigating the antidepressant effects of tDCS as an add-on treatment in MDD [19].

## 2. Materials and Methods

### 2.1. Participants

The participants of this trial were also included and previously described in two studies, with matching inclusion and exclusion criteria, regarding the psychomotor symptoms in MDD in comparison to healthy controls (see Bennabi et al. [18]) and a feasibility study on the application of tDCS to treatment-resistant MDD (see Bennabi et al. [19]). Twenty-three patients (17 females, 6 males, mean age ± SD: 61.8 ± 16.3 years old) meeting Diagnostic and Statistical Manual of Mental Disorders (DSM-IV) criteria for unipolar depression were recruited from the psychiatric wards of the University Hospital of Besançon, France. Patients were required to have a score ≥25 on the MADRS [20] and to meet at least stage II treatment-resistant criteria. Patients diagnosed with bipolar depression, psychotic features, neurological disease, severe organic disease, or undergoing treatment with first-generation antipsychotics (FGA) were excluded from this study. Every patient received escitalopram as antidepressant medication in a constant dosage (10–20 mg/day) for four weeks prior to the experiment. Concomitant medication with benzodiazepines was allowed. The pharmacological treatment was unchanged during these 4 weeks preceding stimulation sessions (add-on treatment) and during the follow-up period. A written informed consent was given by all participants prior to enrollment. Research protocol was approved by the French Committee of Protection of Persons (CPP)-Est-II and was conducted in line with the Declaration of Helsinki.

### 2.2. Experimental Design

The study design was similar to that described by Bennabi et al. [19]: a double-blinded, randomized, sham-controlled trial, aiming to explore the effect of ten sessions of tDCS applied over the left dlPFC on PMR in unipolar depression. Following completion of baseline clinical and experimental measures, subjects were randomly assigned to receive either active or sham tDCS (in addition to their pharmacological treatment), using a computer-generated randomization list with the information stored on a centralized computer (for a full description of the study design, see Bennabi et al. [19].

### 2.3. Transcranial Direct Current Stimulation

Direct current was generated by a battery-driven, constant- current stimulator (“Eldith” stimulator, Ilmenau, Germany), and transmitted by two 5 × 7 cm^2^ saline-soaked synthetic sponge electrodes. The anode was placed over the left dlPFC and the cathode over the contralateral supraorbital area, corresponding to F3 and FP2 according to the international 10–20 EEG System. In the active group, stimulation intensity was delivered at 2 mA for 30 min, twice a day, for 5 days consecutively. For sham stimulation, the procedure was identical, excluding the fact that the current was gradually ramped down to zero, thus leading to the same initial sensations of tDCS. Predefined codes assigned to either real or sham stimulation were used to start the stimulator, allowing a double-blind study design.

### 2.4. Psychiatric Assessment

Depression severity and PMR were evaluated at baseline (T1) and immediately after the end of the treatment (T2) by a trained psychiatrist. Rating scales of depression included the 21-item HDRS, the MADRS and the Beck Depression Inventory (BDI). PMR was evaluated with the Salpêtrière Retardation Rating Scale (SRRS) [21].

### 2.5. Movement Tasks

The tasks used in this trial were modified from our previous studies [15,16,17] and are more precisely described by Bennabi et al. [18]. All participants were seated in a darkened room in front of a large rear projection screen (170 × 230 cm) placed 10 cm beyond the end of the participant’s extended arm. The visual stimuli were back-projected onto the display screen with a video projector placed behind the screen and connected to a computer. The projected visual stimulation was generated using MatLab Pyschotoolbox^®^. One passive infrared reflective marker (diameter: 20 mm) was applied onto a fingertip of the participant’s right hand, and arm movements were recorded using an optoelectronic system (SMART CAPTURE), with six cameras recording movements at a sampling frequency of 120 Hz. The device was calibrated for each participant at the beginning of the experimental sessions. Each participant performed a pointing movement task (PM) and a movement observation task (MO). These tasks were presented in two separate blocks, the PM before the MO.

**Figure 1:**
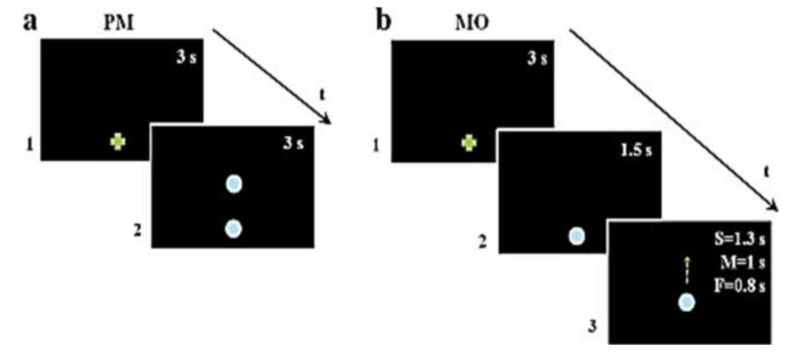
Sequence of visual stimuli. (**a**) In the pointing movement experiment (PM), a green cross appeared on the screen to indicate the starting point. After 3 s, the green cross disappeared and two vertically aligned light blue dots (3.2 cm in diameter with a 51 cm gap between them) were displayed for 3 s. (**b**) In the movement observation experiment (MO), a green cross was displayed to indicate the movement’s starting point. After 3 s, the green cross was replaced by a light blue dot (3.2 cm in diameter). The dot remained at this position for 1.5 s and then started to move vertically upwards for a total of 51 cm of displacement on the screen with three different speeds.

Pointing movement task (PM): This task aimed to measure participants’ natural pointing movements. The kinematic data served as baseline to be compared with arm kinematics after motion observation. A green cross appeared on the screen to indicate the starting point. After 3 s, the green cross disappeared and two vertically aligned light blue dots (diameter: 3.2 cm, with a 51 cm gap between them) were displayed for 3 s. One of the two dots replaced the green cross and the other one was the target for the movement (Figure 1a). Participants performed upwards movements with their right arm in extended position from the given starting point to the target dot at their spontaneous natural speed. The PM was repeated five times, and movement accuracy was not emphasized.

Movement observation task (MO): In this experiment, a green cross was displayed to indicate the movement’s starting point. After 3 s, the green cross was replaced by a light blue dot (diameter: 3.2 cm). The dot remained at this position for 1.5 s and then started to move upwards in a vertical direction with a biological kinematic until it reached 51 cm of displacement. Dot motions randomly displayed three different mean speeds (Vp): slow (S = 28 m/s), medium (M = 43 m/s), and fast (F = 52 m/s) (Figure 1b). Participants accomplished two types of tasks, implicit (i) and explicit (e), which differed only for their instruction. In implicit movement observation (iMO), participants were asked to point at the green cross, to observe the movement of the blue dot, then to wait until the dot reached its final visible position, and finally to point once again to its final position. In explicit movement observation (eMO), the instructions were similar, but participants were requested to imitate (follow) the stimulus motion speed. There were six trials for each dot motion speed resulting in a total of 18 trials. The iMO always preceded the eMO in order to prevent contamination of the implicit movement by the explicit instruction. For both tasks, the beginning of the experiment was preceded by a training phase, which ended when the participant understood the task and correctly accomplished the whole experiment at least twice. Moreover, each participant received verbal feedback from the experimenter during the testing procedure in order to eliminate any confusion about its aim.

### 2.6. Data Analysis

Data processing: Data were low-pass filtered at 5 Hz using a second order Butterworth filter. To define the onset and offset of the movement, we chose a threshold corresponding to 10% of the maximum value of the movement speed profile.

Data analysis: Analyses were performed using MatLab^®^ software. In the PM, data recorded were the reaction time (RT; the time elapsed between the appearance of the two dots and the onset of the participant’s arm movement), the duration (DUR) of the movements, the mean speed (Vmean), the maximum speed (Vmax) and the normalized jerk (the rate of change in acceleration).

In the MO, the RT (i.e., the time difference between the end of the dot motion and the onset of the participant’s movement) and the Vmean were analyzed.

### 2.7. Statistical Analysis

Analyses were performed using Stata Software release 10.1 (StataCorp, College Station, TX, USA). Baseline demographic and clinical data were compared using the Fisher’s exact test (for categorical variables) or the unpaired t-test (for continuous variables). Concerning psychiatric assessments, baseline psychiatric scores were statistically compared using the paired *t*-test or the Wilcoxon signed ranks test. The changes in the scores from baseline were calculated by subtracting baseline scores from the scores at T2. ANCOVA was performed using baseline as covariate and differences from baseline to T2 as dependent variables to assess significant differences between groups after the 10 sessions of tDCS. Concerning motor performance in the PM, iMO and eMO experiments, the kinematic parameters of the two groups (active vs. sham) were statistically compared at baseline using the paired *t*-test or the Wilcoxon signed ranks test. The differences for kinematic parameters evolution in the PM, iMO and eMO experiment were assessed using ANCOVA, including groups (active–sham) as factor and baseline scores as covariate. The significance alpha level was fixed at 0.05.

## 3. Results

Demographic characteristics did not differ across the treatment groups for mean age ± SD (active group: 60.4 ± 12 years; sham group: 59.9 ± 15.4 years; t21 = 0.09, *p* = 0.93), gender (female % active group: 83.3%; sham group: 45.5%; z = 1.33, *p* = 0.09) or educational level (active group: 10.2 ± 2.4 years; sham group: 11.9 ± 3.1 years; t21 = 1.72, *p* = 0.18). The subjects’ clinical characteristics are summarized in Table 1. There were no significant differences in baseline characteristics between active and sham groups (all measures: *p* > 0.05). After ten tDCS sessions, no significant differences were found within the clinical measures after comparison between real treatment and sham stimulation.

### 3.1. Pointing Movement Task (PM)

Motor performances did not differ across the treatment groups before stimulation. Moreover, no significant differences were found within the motor measures after comparison between real treatment and sham stimulation concerning RT, DUR, Vmean, Vmax, and normalized jerk. A complete description of the statistical results is provided in Table 2.

### 3.2. Movement Observation Task (MO)

The MO’s performances are presented for both groups in Table 3, in implicit and explicit conditions. In the iMO task, ANCOVA analysis did not show differences between the active and sham groups on RT and Vmean, regardless of the dot speed.

Similar results were found for kinematics parameters in the eMO task. Regardless of the dot speed, RT and Vmean did not differ between the two groups.

## 4. Discussion

This study explored the psychomotor effects of anodal tDCS to the left dlPFC in treatment-resistant depression, applying the SRRS and an objective assessment method designed to evaluate the kinematic features of movements. The effects of tDCS sessions on mood and depression severity are reported in detail by Bennabi and colleagues [19]. Psychomotor symptoms rated with the SSRS improved by 22.2% in active versus 15.6% in sham stimulation group from baseline after 10 stimulation sessions. However, active stimulation failed to demonstrate any additional effects on this measure when compared with sham. Concerning the kinematic parameters, the analyses did point towards better end-point performances, but revealed no statistically significant differences between the two groups. Notably, at the perceptual and motor levels, the lack of effect on movements duration and smoothness (i.e., normalized jerk) suggests that active tDCS has no effect on the ability to maintain the initial motor plan throughout its course with respect to sham stimulation.

The lack of offline effect of tDCS on motor performance observed in the present study corroborates the findings of two consecutive studies by Loo et al. [11,12], which failed to predict the response to anodal tDCS using the CORE index, a scale specifically designed to quantify the degree of psychomotor impairment. The discrepancies between the studies of Alonzo et al. [13] and D’Urso et al. [14] and our results could be related to the features of the assessment tools. In its broadest sense, the term PMR is used to describe all behavior that depends on both mental and motor processes [22]. The MADRS includes only one item for psychomotor disturbance, and cognitive or motor aspects of agitation and retardation are intermixed. Retardation also appears indirectly in different items concerning fatigue, loss of energy, or lack of concentration. On the contrary, the use of specific rating scales and experimental methods for assessing PMR allow the combination of prolonged clinical observations and the assessment of fine motor performance during task execution (for review, see Bennabi et al. [7]). Moreover, the iMO task assesses automatic and unconscious responses that are not requiring attentional resources [15,16,17].

Besides assessment methods, the absence of an effect of tDCS on motor performance may be related to the patients’ characteristics and stimulation parameters used. Indeed, as a higher level of treatment resistance in the current depressive episode might be inversely related to clinical outcome, this might to some extent have impacted our motor task results [6]. The influence of age on the performance of participants (mean 61.8 ± 16.3 years old) could as well be a confounding factor, since a global slowing in processing stages due to aging [23] may superimpose depression-associated PMR. In addition, the presence of depression biotypes that may be less responsive to non-invasive brain stimulation [24] among our participants could as well be associated with the absence of significant results. Moreover, D’Urso et al. [14] and Alonzo et al. [13] treatment protocols proposed to stimulate patients twice daily with a total of fifteen and twenty sessions, respectively. Therefore, the application of 10 sessions over one week in our study might be considered as rather short. Additionally, scores on a motor task under tDCS influence (online) were not collected in this trial.

From a neurobiological standpoint, PMR has been associated with reduced metabolic activities in the dorsolateral prefrontal cortex and abnormalities in the basal ganglia and dopaminergic pathways [7,10,25]. Some neurobiological findings point to a reduced dopamine synthesis capacity in the striatum, specifically related to PMR in depressed individuals [25]. In healthy subjects, active but not sham stimulation of the frontal areas has been found to produce a significant decrease in [11C] the raclopride binding potential ratio in the striatum, suggesting an increase in extracellular dopamine in a part of the striatum [26]. This could imply that our tDCS study failed to induce up- or down-regulation of the activity in the left dlPFC to modify dopaminergic response from the structures connected with the targeted region, such as the striatum, and thus to initiate indirect dopamine-dependent behavior modification. However, without concomitant brain imaging techniques, these assumptions should be interpreted cautiously at this point.

A limitation of the present study is that the sample size is relatively small and the observation periods are short. Hence, trials with larger samples sizes applying clustering methods could allow the identification of depression subtypes that are more or less responsive to tDCS, as previously described for rTMS [24]. Moreover, all patients were receiving psychotropic medication, and the possibility that this treatment had an influence on our results must be considered. Pharmacological treatments may contribute to improving psychomotor functioning, but might also have disruptive effects, causing sedation or impairment in psychomotor and cognitive function [27]. Benzodiazepines have been associated to disruption of the neurophysiological effects of stimulation, whereas adjunctive antidepressant medication could enhance the therapeutic effects of stimulation [28]. Notwithstanding, during the stimulation protocol, no changes to the patients’ habitual treatment were allowed, and psychotropic medications remained unchanged during the 4 weeks preceding stimulation. Moreover, comparison between online and offline sessions could also lead to a better understanding of tDCS effects. Finally, it would be equally interesting to obtain more knowledge on the effects of tDCS on different areas of the psychomotor functioning. PMR in MDD can lead to disturbances in speech, facial expression, fine motor behavior, gross locomotor activity, or ideation. However, the relationship between all these abnormalities has not yet been elucidated, and it is not clear whether MDD patients with PMR are affected to the same degree in all motor domains.

## 5. Conclusions

Our study revealed a lack of significant effects of tDCS sessions over the left dlPFC on psychomotor functioning in patients suffering from treatment-resistant unipolar depression, suggesting that repeated offline sessions might have no cumulative effects for PMR. Although in the last decade the studies of the therapeutic role of tDCS in depression have grown considerably, leading to promising results in this indication, most of the data focused on clinical measures of depressive symptoms or severity of depression. Profiling the antidepressant effects of tDCS is of particular interest, as it may help to identify target populations. However, the present findings did not confirm an effect of tDCS, neither on motor planning nor on response selection in this indication.

## Figures and Tables

**Table 1:** Clinical (mean ± SD) data at baseline and T2 for active (*N* = 12) and sham group (*N* = 11).

	Baseline		T2			
Clinical Scales	Active	Sham	Active	Sham	F (1.22)	*p*
MADRS	29.2 ± 4.0	34.2 ± 7.5	21.3 ± 8.0	28.4 ± 11	0.92	0.35
HDRS 21	22.7 ± 5.3	24.8 ± 5.6	15.25 ± 6.1	19.8 ± 7.5	2.01	0.17
BDI	18.7 ± 6.1	19.5 ± 7.3	14 ± 5.7	15.1 ± 6.4	0.25	0.62
SRRS	27 ± 7.1	28.9 ± 10.6	21 ± 5.5	24.4 ± 11.8	1.52	0.28

BDI: Beck Depression Inventory, HDRS 21: Hamilton Depression Rating Scale 21 items; MADRS: Montgomery and Asberg Depression Rating Scale; SRRS: Salpêtrière Retardation Rating Scale.

**Table 2:** Difference in kinematics parameters (mean ± SD) between active (*N* = 12) and sham (*N* = 11) groups at baseline and their evolution after stimulation.

	Baseline			T2-Baseline		Groups	
	Active	Sham	*p*	Active	Sham	F (1.22)	*p*
RT(s)	0.45 ± 0.18	0.48 ± 0.24	0.61	0.01 ± 0.2	−0.01 ± 0.2	0.01	0.92
DUR(s)	0.81 ± 0.23	0.82 ± 0.24	0.63	−0.03 ± 0.2	−0.05 ± 0.2	0.03	0.86
Vmean(m/s)	0.63 ± 0.17	0.6 ± 0.17	0.46	−0.001 ± 0.1	0.03 ± 0.14	0.2	0.66
Vmax(m/s)	1.25 ± 0.38	1.14 ± 0.3	0.29	−0.04 ± 0.3	0.04 ± 0.2	0.03	0.87
Jerk	27.39 ± 8.31	28.75 ± 7.22	0.68	−3.09 ± 2.8	−5.01 ± 3.1	0.07	0.79

RT: reaction time; DUR: duration; V_mean_: mean speed; V_max_: maximum speed.

**Table 3:** Difference in kinematics parameters (mean ± SD) between active (*N* = 12) and sham (*N* = 11) groups at baseline and their evolution after stimulation in implicit and explicit movement observation tasks.

		Baseline			T2-Baseline		Groups	
		Active	Sham	*p*	Active	Sham	F (1.22)	*p*
iMO	RT (s)							
	Slow	−0.14 ± 0.39	−0.22 ± 0.44	0.63	0.29 ± 0.45	0.28 ± 0.45	0.33	0.57
	Medium	−0.07 ± 0.36	−0.07 ± 0.28	0.98	0.20 ± 0.36	0.18 ± 0.25	0.06	0.81
	Fast	0.05 ± 0.36	0.01 ± 0.3	0.75	0.22 ± 0.33	0.20 ± 0.44	0.29	0.6
	V_mean_ (m/s)							
	Slow	0.56 ± 0.1	0.53 ± 0.17	0.67	−0.021 ± 0.08	−0.01 ± 0.12	0.05	0.82
	Medium	0.56 ± 0.1	0.53 ± 0.13	0.66	−0.02 ± 0.11	0.02 ± 0.10	0.61	0.44
	Fast	0.59 ± 0.11	0.59 ± 0.19	0.96	0 ± 0.08	0 ± 0.12	0.07	0.8
eMO	RT (s)							
	Slow	−0.02 ± 0.38	0.11 ± 0.28	0.51	0.13 ± 0.14	−0.03 ± 0.38	1.59	0.22
	Medium	0.13 ± 0.32	0.18 ± 0.26	1	0.06 ± 0.19	0 ± 0.20	0.61	0.44
	Fast	0.17 ± 0.27	0.26 ± 0.27	0.53	0.09 ± 0.15	−0.03 ± 0.21	2.12	0.16
	V_mean_ (m/s)							
	Slow	0.42 ± 0.07	0.41 ± 0.09	0.78	−0.02 ± 0.07	−0.01 ± 0.08	0.07	0.79
	Medium	0.48 ± 0.06	0.47 ± 0.11	0.75	−0.03 ± 0.09	−0.01 ± 0.07	0.1	0.75
	Fast	0.55 ± 0.12	0.53 ± 0.13	0.71	0.02 ± 0.08	−0.01 ± 0.09	2.17	0.16

iMO: implicit movement observation; eMO: explicit movement observation; RT: reaction time; V_mean_: mean speed.
